# Monitoring Arthropods in Azorean Agroecosystems: the project AGRO-ECOSERVICES

**DOI:** 10.3897/BDJ.9.e77548

**Published:** 2021-12-09

**Authors:** Paulo A. V. Borges, Rui Nunes, Lucas Lamelas-López, Enésima Pereira, Ricardo Costa, Paulo Monjardino, David H. Lopes, António Onofre Soares, Artur Gil, François Rigal, Marco Ferrante, Gabor L. Lövei

**Affiliations:** 1 cE3c – Centre for Ecology, Evolution and Environmental Changes/ Azorean Biodiversity Group and Universidade dos Açores, Rua Capitão João d’Ávila, São Pedro, 9700-042, Angra do Heroísmo, Azores, Portugal cE3c – Centre for Ecology, Evolution and Environmental Changes/ Azorean Biodiversity Group and Universidade dos Açores, Rua Capitão João d’Ávila, São Pedro, 9700-042 Angra do Heroísmo, Azores Portugal; 2 CBA – Biotechnology Centre of Azores, University of Azores, Faculty of Agriculture and Environment, Rua Capitão João D'Avila, 9701-042, Angra do Heroísmo, Azores, Portugal CBA – Biotechnology Centre of Azores, University of Azores, Faculty of Agriculture and Environment, Rua Capitão João D'Avila, 9701-042 Angra do Heroísmo, Azores Portugal; 3 cE3c – Centre for Ecology, Evolution and Environmental Changes/ Azorean Biodiversity Group and Universidade dos Açores, Faculty of Sciences and Technology, 9500-321, Ponta Delgada, Azores, Portugal cE3c – Centre for Ecology, Evolution and Environmental Changes/ Azorean Biodiversity Group and Universidade dos Açores, Faculty of Sciences and Technology, 9500-321 Ponta Delgada, Azores Portugal; 4 IVAR - Research Institute in Volcanology and Risk Assessment | University of the Azores, Ponta Delgada, Azores, Portugal IVAR - Research Institute in Volcanology and Risk Assessment | University of the Azores Ponta Delgada, Azores Portugal; 5 CNRS - Université de Pau et des Pays de l’Adour, Institut des Sciences Analytiques et de Physico-Chimie pour l’Environnement et les Matériaux, E2S, UMR 5254, BP, Pau Cedex, Pau, France CNRS - Université de Pau et des Pays de l’Adour, Institut des Sciences Analytiques et de Physico-Chimie pour l’Environnement et les Matériaux, E2S, UMR 5254, BP, Pau Cedex Pau France; 6 Department of Agroecology, Aarhus University, Flakkebjerg Research Centre, Forsoegsvej 1, DK-4200, Slagelse, Denmark Department of Agroecology, Aarhus University, Flakkebjerg Research Centre, Forsoegsvej 1, DK-4200 Slagelse Denmark

**Keywords:** Active Aerial Searching (AAS), citrus, dataset, invertebrates, island diversity, Macaronesia, maize, occurrence, orchards, pitfall traps, vineyards.

## Abstract

**Background:**

The data we present are part of the AGRO-ECOSERVICES project (Assessing ecosystem services and disservices provided by arthropod species in Azorean agroecosystems). The project aims to evaluate the relative importance of native and non-native organisms as ecosystem services (ES) and disservices (ED) providers, by combining novel, direct and quantitative tools for monitoring agro-biodiversity. Ecosystem services include evaluation of natural pest control by predation, seed predation on weed plants, pollination, decomposition and ecosystem disservices, herbivory and seed predation on crop plants. Active Aerial Searching (AAS) (only in maize-fields) and pitfall traps were used to sample the arthropod biodiversity (predatory spiders, true-bugs and beetles and main insect pests) on four agricultural habitats of Terceira Island, namely citrus orchards, low and high elevation maize fields and vineyards.

**New information:**

We provided an inventory of all arthropods recorded in four Azorean agroecosystems (citrus orchards, low and high elevation maize fields and vineyards) from Terceira Island. A total of 50412 specimens were collected, belonging to four classes, 20 orders, 81 families and 200 identified species of arthropods. A total of 127 species are considered introduced (n = 22646) and 69 native non-endemic (n = 24117). Four endemic species were recorded with very few specimens (n = 14) and 3635 specimens belong to unidentified taxa recorded only at genus or family level. Five species are new records for Terceira Island, with *Lagriahirta* (Linnaeus, 1758) (Coleoptera, Tenebrionidae) being also a new record for the Azores. This publication contributes to a better knowledge of the arthropods communities present in agro-ecosystems of Terceira Island and will serve as a baseline for future monitoring schemes targeting the long-term change in arthropod diversity and abundance.

## Introduction

Land-use transformation with associated habitat degradation, is one of the major drivers of biodiversity loss worldwide (Vitousek et al. 1997, Barnosky et al. 2011, Borges et al. 2019a, Harvey et al. 2020). In the case of Azores, since Portuguese colonisation in the 15^th^ century, the original landscape has suffered severe transformations, with the replacement of native forests by exotic tree plantations, pastures, agricultural and urban areas (Gaspar et al. 2008, Borges et al. 2019a, Borges et al. 2019b, Norder et al. 2020).

However, although exotic species have a competitive advantage to colonise new human-altered habitats given that their tolerance to wide range of environmental conditions and habitats (e.g. generalist behaviour) (Rigal et al. 2017), these non-natural habitats also offer opportunities to native biota (McKinney and Lockwood 1999, Blackburn et al. 2004, Sax 2008, Tsafack et al. 2021).

Many species were also introduced because of human settlement (Frutuoso 2011). The current remnants of native forests represent less than 5% of the total area of the archipelago (Gaspar et al. 2008). Currently, the Azorean economy depends greatly on agroecosystems (Gil et al. 2017). Agrosecoystems with the largest area are pastures, followed by maize, with the two crops usually grown in rotation. Due to their long co-existence and close taxonomic relationship between pastures and maize (both are grasses), several pests interact with both crops all year round (P. Monjardino, pers. observ.). These interactions need to be further understood, because of ongoing current significant yield losses in both agroecosystems (P. Monjardino, pers. observ.). Vineyards and citrus orchards are amongst the most important crops on the Azores. Both crops have significant pest and disease problems due to the benign environmental conditions and to improper cultural practices (Lopes et al. 2009).

Azorean terrestrial arthropod fauna have been extensively surveyed in the last two decades. Although most surveys have been conducted in native forests (e.g. Borges et al. 2005, Ribeiro et al. 2005, Borges et al. 2006), several also included anthropogenic habitats, as exotic forest plantations, pastures for cattle grazing and other agricultural areas (Cardoso et al. 2009, Florencio et al. 2015, Rigal et al. 2017, Marcelino et al. 2021, Tsafack et al. 2021).

In 2019 and 2020, we started the project “Assessing Ecosystem Services and Disservices provided by Arthropod species in Azorean Agroecosystems” (AGRO-ECOSERVICES). This project aims to: (i) initiate the monitoring of terrestrial arthropods in agricultural habitats, (ii) implement novel, direct and quantitative tools to quantify ecosystem services (ES) and disservices (ED) and (iii) evaluate the relative importance of native and non-native organisms as ES/ED providers.

Arthropods, especially insects, support ecosystem stability and functioning (Allan et al. 2015, Bennett et al. 2015). Due to their high species richness and abundance, as well as their importance for several ES and ED (Zhang et al. 2007, Ameixa et al. 2018, Noriega et al. 2018, Ecosystem Services 2019), arthropods play a key role in all terrestrial ecosystems. Evaluating the total effect of arthropods that are providers of both ES and ED is challenging (Shapiro and Báldi 2014). For example, when they prey on pests, generalist predators provide biological control, an ES valued at $400 billion/y (Costanza et al. 1997), while their intraguild predation (Lövei and Ferrante 2017) constitutes an ED. A second great challenge is to assess the role of native vs. exotic biodiversity in providing ES/ED, which is essential to manage sustainable landscapes and an important frontier in theoretical ecology. Exotic species often alter ecological processes and cause severe biodiversity loss (Simberloff et al. 2013). Nevertheless, these species may also provide ES: alien plants can increase microbial activity (Vilà et al. 2011), introduced natural enemies can control pests (Heimpel and Mills 2017) or provide ecological “insurance” after the decline of native species (Stavert et al. 2018).

Oceanic islands have a high proportion of endemic species, being very sensitive to biotic disturbance, such as invasions and land-use changes (Stachowicz and Tilman 2005, Kier et al. 2009) - the perfect setting to test the response of ecological communities to disturbance and its effects on ecosystem processes. Several factors contribute to arthropod decline in the Azores (Borges et al. 2019b), including native forest destruction (Triantis et al. 2010), lack of connectivity between forest patches (Aparício et al. 2018) and climate change (Ferreira et al. 2016).

This publication contributes not only to a better knowledge of the arthropods present in agroecosystems of Terceira Island, but will also contribute as a baseline for future monitoring schemes in Azorean agroecosystems targeting the long-term change in arthropod diversity and abundance.

## General description

### Purpose

To provide an arthropod inventory of agro-ecosystems from Terceira Island (Azores), based on data collected in four agro-ecosystems, citrus orchards, low and high elevation maize fields and vineyards. This study will contribute to a better knowledge of the arthropods present in agro-ecosystems and will serve as a baseline for future monitoring schemes in Azorean agro-ecosystems targeting the long-term change in arthropod diversity and abundance.

### Additional information

The study was conducted between July 2019 and September 2021 in Terceira Island. Active Aerial Searching (only in maize-fields) and pitfall traps were used to sample the arthropod biodiversity (pollinators and predatory spiders, true-bugs and beetles and main insect pests) on four agricultural habitats, namely citrus orchards, vineyards, low elevation maize fields and high elevation maize fields. Information on ecosystem services (ES) and disservices (ED) providers will be the subject of another publication.

## Project description

### Title

AgEcSe- AGRO-ECOSERVICES - Assessing ecosystem services and disservices provided by arthropod species in Azorean Agroecosystems (ACORES-01-0145-FEDER-000073)

### Personnel

Project leaders: Paulo A. V. Borges and António Onofre Soares

Team members: Marco Ferrante, Artur Gil, Marco Girardello, David H. Lopes, Paulo Monjardino, Rui Nunes.

External Consultants: Sven Bacher, Gabor Lövei, François Rigal

Parataxonomists: Jonne Bonnet, Ricardo Costa, Rui Nunes

Darwin Core Database management: Paulo A. V. Borges, Lucas Lamelas-López, Enésima Pereira

### Study area description

**Figure 1.**
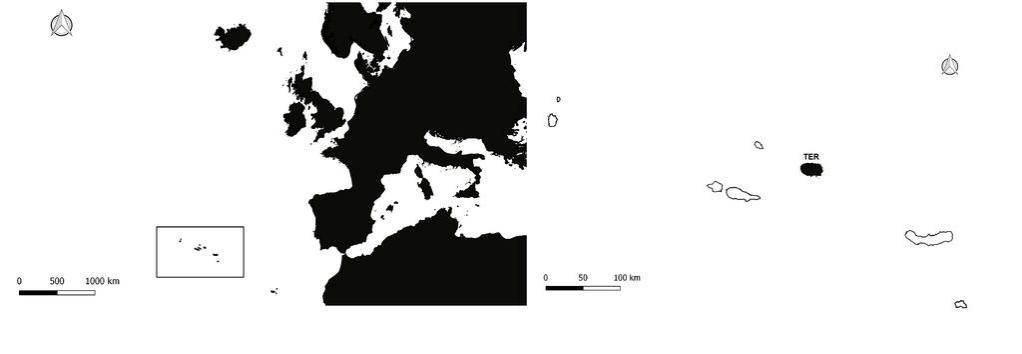
Map of the Azores Archipelago location in mid-Atlantic with the studied island TER - Terceira, marked in black (Credit: Enésima Pereira).

Terceira Island (area: 400.2 km²; elevation: 1021 m a.s.l.) is located in the central group of the Azores Archipelago (North Atlantic), roughly at 38.638 N and -27.0150 W (Fig. 1). Similar to all islands in Azores, Terceira is volcanic and of recent origin (0.4 Ma, see Florencio et al. 2021). The climate is temperate oceanic, with regular and abundant rainfall, high levels of relative humidity and persistent winds, mainly during the winter and autumn seasons.

### Design description

**Figure 2.**
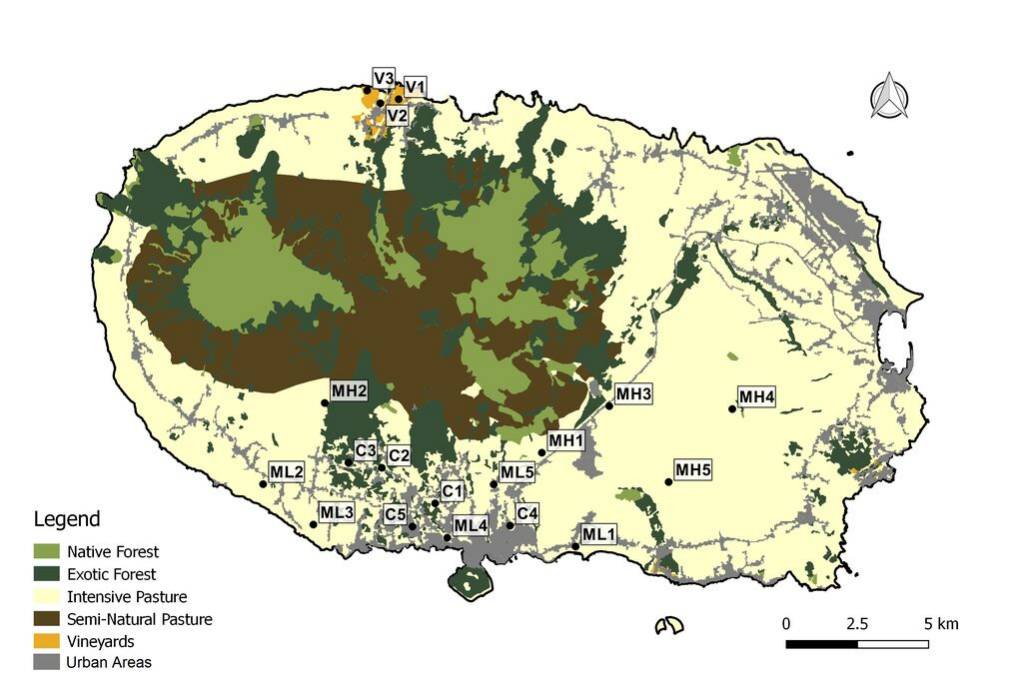
Map of the study area (Terceira Island, Azores). Codes of sites as in Table 1. Maize fields are located in intensive pasture since they are only operating in summer, with the two crops usually grown in rotation (Land-use data extracted from Cruz et al. 2007).

The sampled habitats included citrus orchards, vineyards and low elevation maize fields, all located at low elevation areas and high elevation maize fields (Fig. 2, Table 1). The two types of maize fields differ not only in the elevation, but principally in crop management, the low elevation being an annual rotation of maize and Italian ryegrass and the high elevation (located at intermediate elevation in the Island) being a perennial rotation of maize and perennial ryegrass.

### Funding

This work was financed by FEDER (European Regional Development Fund) in 85% and by Azorean Public funds by 15% through the Operational Program Azores 2020, under the project AGRO-ECOSERVICES (ACORES-01-0145-FEDER-000073).

## Sampling methods

### Study extent

**Figure 3.**
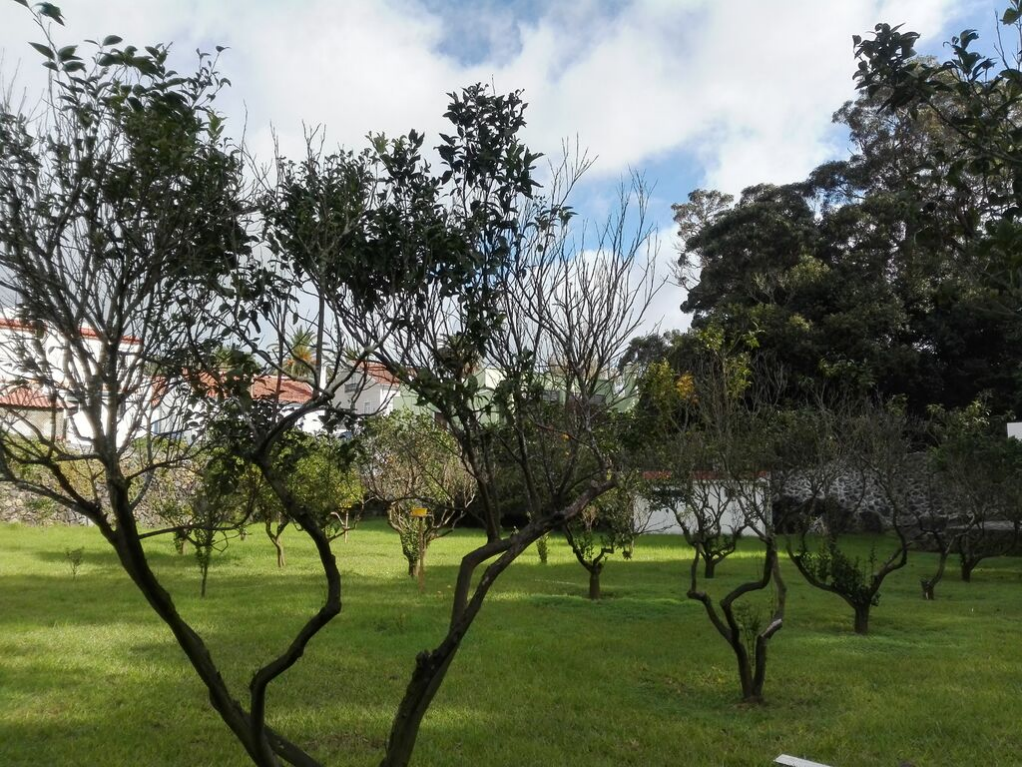
A citrus orchard in Terceira Island (C5 - S. Carlos) (Credit: Rui Nunes).

**Figure 4.**
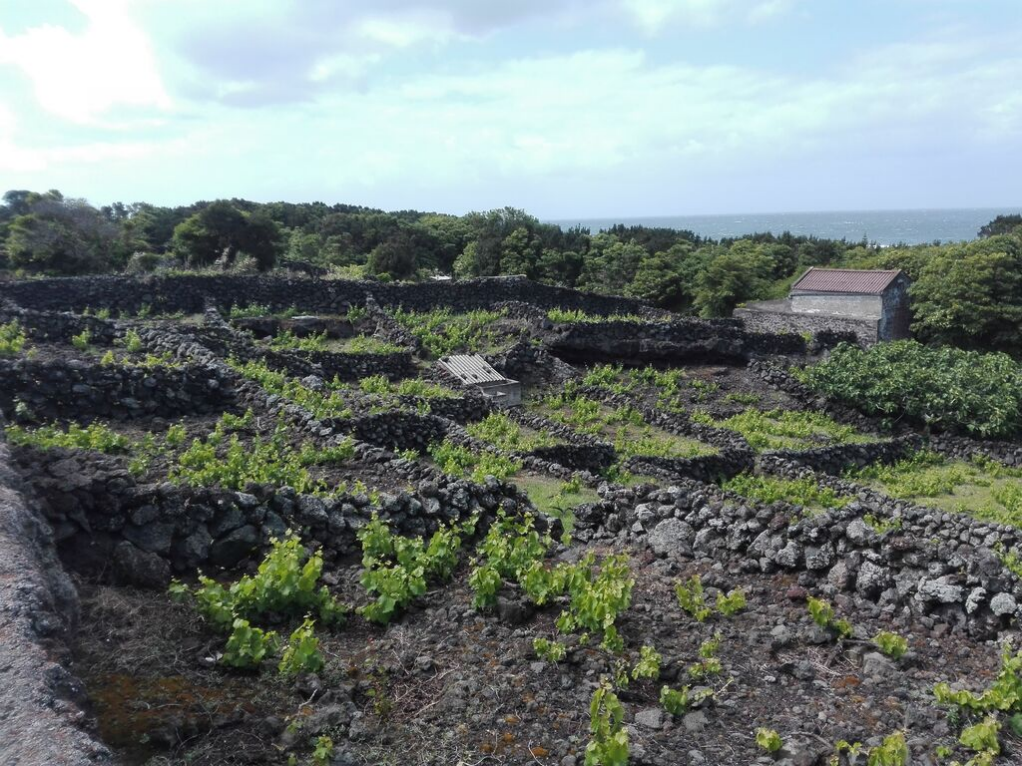
The vineyards in Terceira Island (V3 - Biscoitos) (Credit: Rui Nunes).

**Figure 5.**
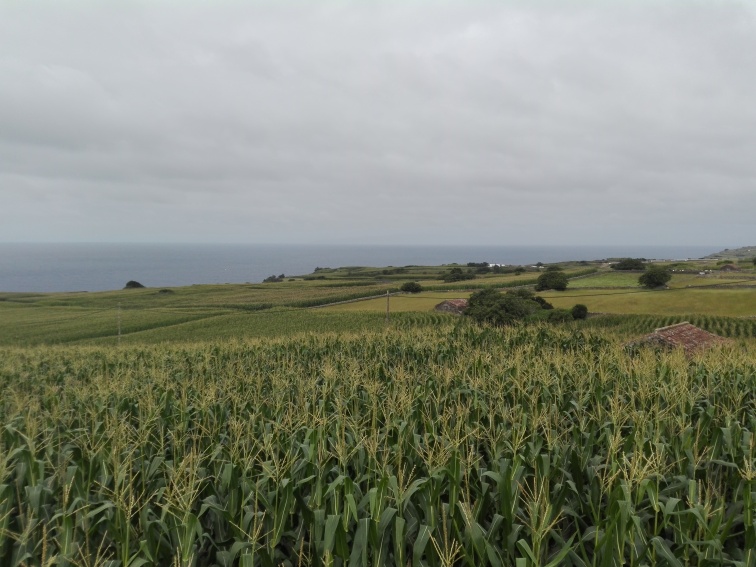
A low elevation maize field in Terceira Island (ML3 - S. Mateus) (Credit: Rui Nunes).

**Figure 6.**
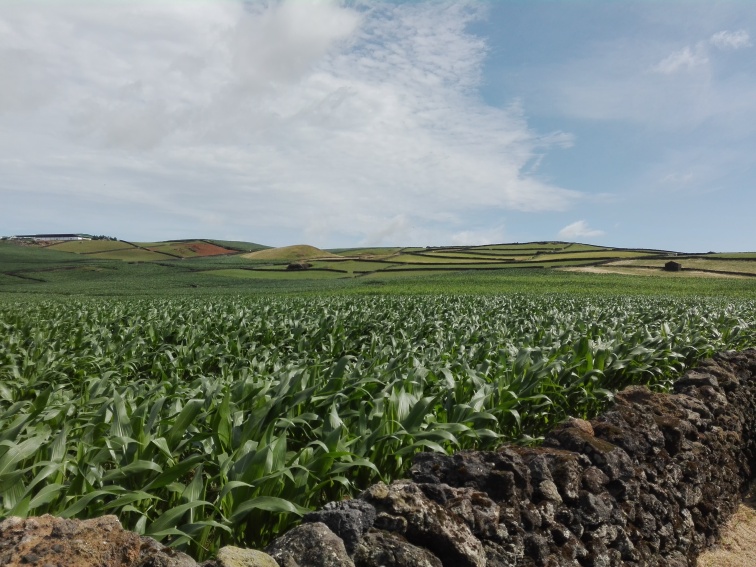
A high elevation maize field in Terceira Island (MH5 -Poejo) (Credit: Rui Nunes).

The study was conducted in four agro-ecosystems of Terceira Island (Fig. 2): citrus orchards (Fig. 3), vineyards (Fig. 4), low elevation maize fields (Fig. 5) and high elevation maize fields (Fig. 6). Five citrus orchards were selected, located at low elevation areas. Ten maize fields, five of which are located inland at higher elevation and five other closer to the coast in low elevation areas. Finally, three vineyards located on the coast, north of the Island were sampled (see also Table 1).

### Sampling description

Active Aerial Searching (AAS) and pitfall traps were used to sample arthropod diversity. The following main functional groups were collected: predatory arthropods (mostly spiders, true-bugs, beetles and bugs), phytophagous insects and saprophagous arthropods (mostly millipedes and beetles).

AAS consists in picking arthropods found above knee-level by hand, using forceps, pooter or brush and immediately transferring them into vials containing ethanol 96%. It was implemented in five low- and five high-elevation maize fields. Four 1-hour samples were obtained during the night when the main predators are more active. Sampling was performed in the summer when the maize plants were at maximum development. Samples were taken by Paulo A. V. Borges and Rui Nunes (two hours each per site).

**Figure 7.**
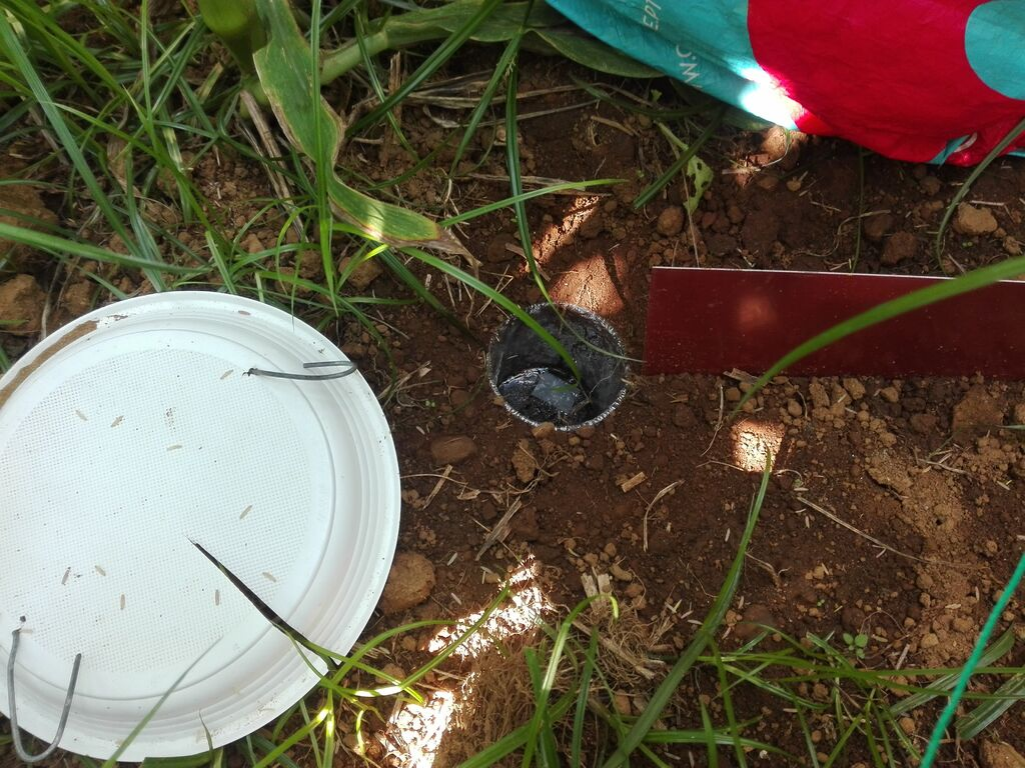
Detail of a pitfall trap (standard 330 ml plastic cups, 8 cm wide at the top and approximately 12 cm deep) (Credit: Rui Nunes).

Pitfall traps were standard 330 ml plastic cups, 8 cm wide at the top and approximately 12 cm deep - European standard plastic cups (Fig. 7), partially filled with propylene glycol. The traps were deployed for 14 consecutive days.

**Figure 8.**
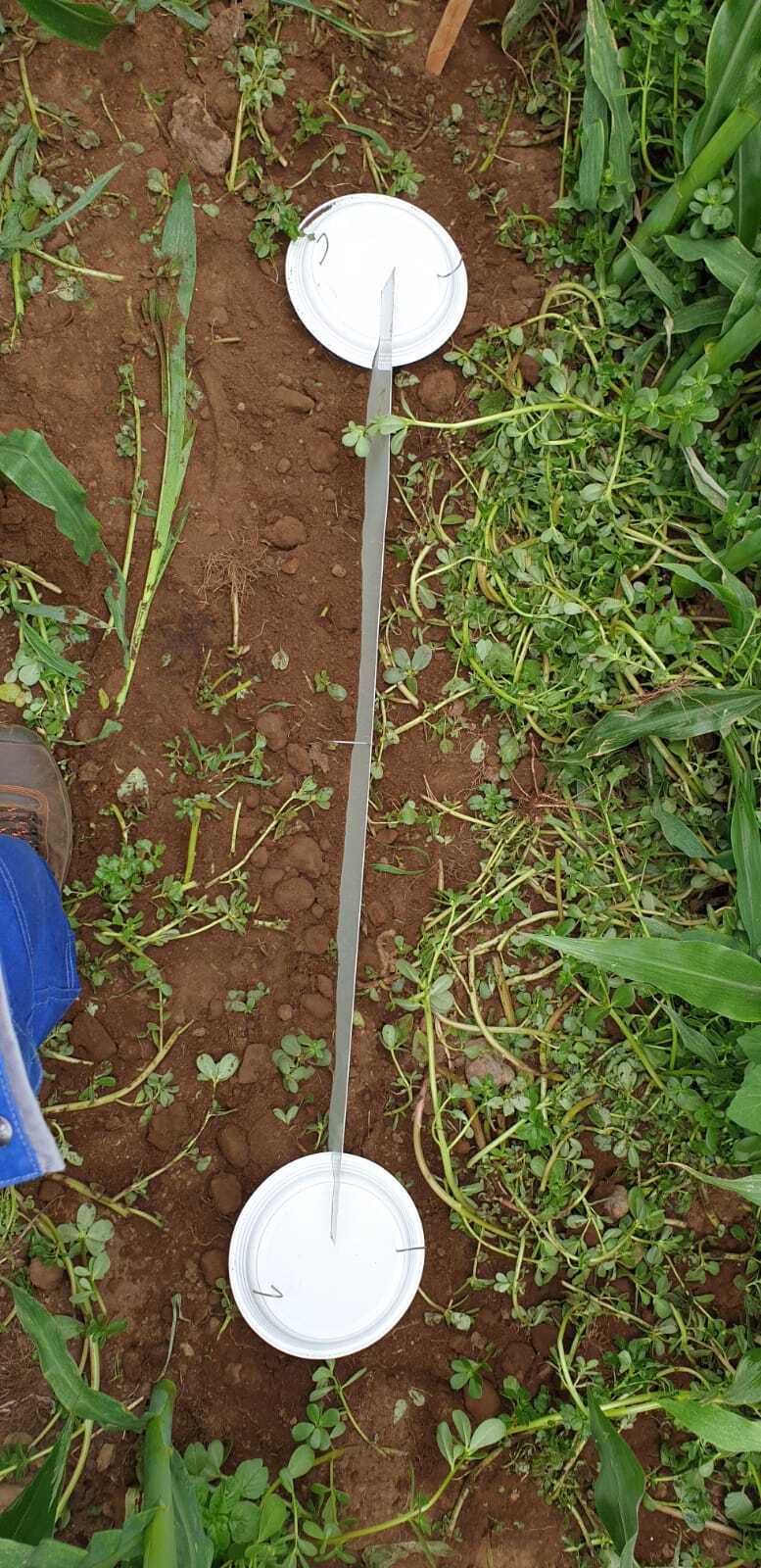
Pitfall traps used in citrus orchards and maize fields (sets of two connected with a grid) (Credit: Rui Nunes).

In each of five citrus orchards and six (of ten available) maize fields (three in low- and three in high-elevation areas), 16 pitfall traps organised in sets of two connected with a grid (Fig. 8) were deployed, along a transect, from the point closest to the crop edge. The eight sets of two pitfall traps were separated by at least 10 metres. A total of 80 and 96 pitfall traps were deployed on citrus orchards and maize fields, respectively.

For vineyards, a different strategy had to be followed since Azorean vineyards are formed by small rocky enclosures (between 6-20 m^2^) (Fig. 4) and pitfall traps were deployed in the interior of these enclosures. Following a transect, a total of 144 individual pitfall traps were deployed in three vineyards (48 in each site).

Sampling methods used in citrus and vineyards (pitfall traps) only provide information on the soil-related arthropods; most of crop insect pests (canopy associated species) are not sampled by this sampling technique.

### Quality control

All sampled specimens were first sorted by trained paratoxonomists (Jonne Bonnet, Ricardo Costa, Rui Nunes). All specimens were allocated to a taxonomic species by Paulo A. V. Borges. Juveniles were also included in the data presented in this paper since the low diversity of species in Azores allows their reliable identification. Colonisation status for each identified species is based on Borges et al. 2010 (END - Endemic; NAT - native non-endemic; INTR -introduced).

### Step description

A reference collection for Azorean arthropods (deposited at the Dalberto Teixeira Pombo Insect Collection, University of Azores) started to be prepared in 1999 by one of us (PAVB) and many taxonomists contributed since then in the identification of species. For all the specimens for which adequate identification was not possible, a new "morphospecies code" was created.

## Geographic coverage

### Description

Terceira Island, Azores, Portugal.

### Coordinates

38.638 and 38.814 Latitude; -27.394 and -27.0150 Longitude.

## Taxonomic coverage

### Description

The following classes and orders of arthropods are covered: Arachnida: Araneae, Opiliones, Pseudoscorpiones; Chilopoda: Geophilomorpha, Lithobiomorpha, Scolopendromorpha, Scutigeromorpha; Diplopoda: Chordeumatida, Julida, Polydesmida; and Insecta: Archaeognatha, Coleoptera, Dermaptera, Hemiptera, Hymenoptera, Lepidoptera, Neuroptera, Orthoptera, Psocoptera, Thysanoptera.

### Taxa included

**Table:** 

Rank	Scientific Name	Common Name
class	Araneae	Spiders
class	Opiliones	Opilions
class	Pseudoscorpiones	Pseudoscorpions
class	Diplopoda	Millipedes
class	Chilopoda	Centipedes
order	Archaeognatha	Bristletails
order	Dermaptera	Earwigs
order	Orthoptera	Crickets, Grasshoppers
order	Psocoptera	Barklice
order	Thysanoptera	Thrips
order	Hemiptera	Bugs
order	Neuroptera	Lacewings
order	Coleoptera	Beetles
order	Hymenoptera	Ants
order	Lepidoptera	Moths

## Traits coverage

No data available.

## Temporal coverage

### Notes

16 July 2019 to 9 June 2021

## Collection data

### Collection name

Entomoteca Dalberto Teixeira Pombo at University of Azores

### Collection identifier

DTP

### Specimen preservation method

All specimens were preserved in 96% ethanol.

### Curatorial unit

Dalberto Teixeira Pombo insect collection at the University of the Azores (Curator: Paulo A. V. Borges)

## Usage licence

### Usage licence

Creative Commons Public Domain Waiver (CC-Zero)

## Data resources

### Data package title

Monitoring Arthropods in Azorean Agroecosystems: the project AGRO-ECOSERVICES (AgEcSe)

### Resource link

https://www.gbif.org/dataset/822f3765-6950-40c5-9353-1f335599007c

### Alternative identifiers

https://doi.org/10.15468/mvtmyx

### Number of data sets

1

### Data set 1.

#### Data set name

Monitoring Arthropods in Azorean Agroecosystems: the project AGRO-ECOSERVICES

#### Data format

Darwin Core Archive

#### Number of columns

56

#### Download URL

http://ipt.gbif.pt/ipt/resource?r=arthropods_agroecoservices

#### Data format version

version 1.10

#### Description

The dataset is available on the Global Biodiversity Information Facility platform, GBIF (Borges et al. 2021). The following data table includes all the records for which a taxonomic identification of the species was possible. The dataset submitted to GBIF is structured as a sample event dataset, with two tables: event (as core) and occurrences (abundance data). The data in this sampling event resource have been published as a Darwin Core Archive (DwCA), which is a standardised format for sharing biodiversity data as a set of one or more data tables. The core data file contains 358 records (eventID) and the occurrences file 5134 records (occurrenceID). This IPT (Integrated Publishing Toolkit) archives the data and thus serves as the data repository. The data and resource metadata are available for download from Borges et al. (2021).

**Data set 1.** 

Column label	Column description
Table of Sampling Events	Table with sampling events data (beginning of table).
eventID	Identifier of the events, unique for the dataset.
stateProvince	Name of the region of the sampling site.
islandGroup	Name of archipelago.
island	Name of the island.
country	Country of the sampling site.
countryCode	ISO code of the country of the sampling site.
municipality	Municipality of the sampling site.
decimalLongitude	Approximate centre point decimal longitude of the field site in GPS coordinates.
decimalLatitude	Approximate centre point decimal latitude of the field site in GPS coordinates.
geodeticDatum	The ellipsoid, geodetic datum or spatial reference system (SRS) upon which the geographic coordinates given in decimalLatitude and decimalLongitude are based.
coordinateUncertaintyInMetres	Uncertainty of the coordinates of the centre of the sampling plot.
coordinatePrecision	Precision of the coordinates.
georeferenceSources	A list (concatenated and separated) of maps, gazetteers or other resources used to georeference the Location, described specifically enough to allow anyone in the future to use the same resources.
locationID	Identifier of the location.
fieldNumber	Code of the sample
locality	Name of the locality.
minimumElevationInMetres	The lower limit of the range of elevation (altitude, usually above sea level), in metres.
habitat	The habitat of the sample.
year	Year of the event.
month	Month of the event.
day	Day of the event.
samplingEffort	The amount of effort expended during an Event.
eventDate	Date or date range the record was collected.
samplingProtocol	The sampling protocol used to capture the species.
Occurrence Table	Table with species abundance data (beginning of new table).
eventID	Identifier of the events, unique for the dataset.
type	Type of the record, as defined by the Public Core standard.
licence	Reference to the licence under which the record is published.
institutionID	The identity of the institution publishing the data.
institutionCode	The code of the institution publishing the data.
collectionID	The identity of the collection publishing the data.
collectionCode	The code of the collection where the specimens are conserved.
datasetName	Name of the dataset.
basisOfRecord	The nature of the data record.
occurrenceID	Identifier of the record, coded as a global unique identifier.
recordedBy	A list (concatenated and separated) of names of people, groups or organisations who performed the sampling in the field.
identifiedBy	A list (concatenated and separated) of names of people, groups or organisations who assigned the Taxon to the subject.
dateIdentified	The date on which the subject was determined as representing the Taxon.
organismQuantity	A number or enumeration value for the quantity of organisms.
organismQuantityType	The type of quantification system used for the quantity of organisms.
sex	The sex and quantity of the individuals captured.
lifeStage	The life stage of the organisms captured.
scientificName	Complete scientific name including author and year.
scientificNameAuthorship	Name of the author of the lowest taxon rank included in the record.
kingdom	Kingdom name.
phylum	Phylum name.
class	Class name.
order	Order name.
family	Family name.
genus	Genus name.
specificEpithet	Specific epithet.
infraspecificEpithet	Infrapecific epithet.
taxonRank	Lowest taxonomic rank of the record.
establishmentMeans	The process of establishment of the species in the location, using a controlled vocabulary: 'native', 'introduced', 'endemic', "unknown".
identificationRemarks	Information about morphospecies identification (code in Dalberto Teixeira Pombo Collection).

## Additional information

We collected a total of 50412 specimens, belonging to four classes, 20 orders and 81 families of arthropods. A total of 127 species are considered introduced (n = 22646) and 69 native non-endemic (n = 24117). Four endemic species were recorded with very few specimens (n = 14) and 3635 specimens belong to unidentified taxa recorded only at genus or family level.

Arachnids belonged to three orders, Araneae being the most abundant (95% of arachnid specimens belonged to this order). Chilopoda and Diplopoda classes recorded four and three orders, being Lithobiomorpha and Julida, respectively, the most abundant. Insecta was the most abundant class (n = 39590) recorded in the studied agro-ecosystems, with Coleoptera the most abundant order (38% of specimens).

A total of 200 species were identified (Table 2) and an additional 73 morphospecies need proper identification, totalling potentially 273 species (see Suppl. material 1).

The five most abundant species account for 64% of all identified specimens and include two ant species: *Lasiusgrandis* Forel, 1909 (Hymenoptera: Formicidae) (n = 15876) and *Tetramoriumcaespitum* (Linnaeus, 1758) (Hymenoptera: Formicidae) (n = 3309), the ground-beetle *Pseudoophonusrufipes* (De Geer, 1774) (Coleoptera, Carabidae (n = 7131), the millipede (Diplopoda: Julida) *Ommatoiulusmoreleti* (Lucas, 1860) (n = 2213) and the cricket (Orthoptera: Gryllidae) *Eumodicogryllusbordigalensis* (Latreille, 1804) (n = 1561).

Within the non-identified morphospecies, the most abundant taxa was a millipede (MF 1006) with 1959 specimens mostly sampled in high elevation maize fields (see Suppl. material 1).

Considering only identified species, a total of 10062 (21.48%), 7622 (16.27%), 16390 (34.99%) and 12763 (27.27%) specimens were collected and identified at species level in citrus orchards, low elevation maize fields, high elevation maize fields and vineyards, respectively (Table 2).

The most abundant species in vineyards were the native ant *Lasiusgrandis* (n = 10283), the introduced spider *Zodarionatlanticum* Pekár & Cardoso, 2005 (n = 934) and the native ant *Tetramoriumcaespitum* (n = 327) (Table 2).

The most abundant species in citrus orchards were the native ant *L.grandis* (n = 3058), the introduced millipede *Ommatoiulusmoreleti* (n = 1740) and the native ant *T.caespitum* (n = 1329) (Table 2).

The most abundant species in low elevation maize fields were also ants, *L.grandis* (n = 1444) and *T.caespitum* (n = 1202), followed by the exotic beetle *Typhaeastercorea* (Linnaeus, 1758) (n = 642) and the mirid bug *Trigonotyluscaelestialium* (Kirkaldy, 1902) (n = 493) (Table 2).

Finally, the most abundant species in high elevation maize fields were the introduced ground-beetle *Pseudoophonusrufipes* (n = 6995), the introduced cricket *Eumodicogryllusbordigalensis* (n = 1559), the two rove-beetles *Amischaanalis* (Gravenhorst, 1802) (n = 1321) and *Rugilusorbiculatus* (Paykull, 1789) (757) and also the ant *L.grandis* (n =1091). Two spiders usually very abundant in intensive pastures are also relatively abundant, *Oedothoraxfuscus* (Blackwall, 1834) (n = 577) and *Erigonedentipalpis* (Wider, 1834) (n = 484) (Table 2).

Although the introduced species potentially have the ability to colonise and spread in human-disturbed habitats (e.g. Rigal et al. 2017), our results showed that Azorean agroecosystems represent habitat opportunities for native arthropods. Some of the most abundant species are generalist predators with omnivorous behaviour, like the ants and the ground-beetle *P.rufipes*. Remarkable was the high abundance of the predatory spider *Z.atlanticum* in vineyards that feed on ants and may act as an ED provider. Most other predators potentially provide an ES to the Azorean agroecosystem habitats, particularly in maize fields and vineyards, through biological control of pests (e.g. Heimpel and Mills 2017). Introduced species can also affect native species of arthropods, for example, through opportunistic predation. However, introduced species may also supplement the functional traits lost after the decline of native species in these habitats (e.g. Stavert et al. 2018).

Five species are new records for Terceira Island: three beetles (Coleoptera), one millipede (Diplopoda: Julida) and one true bug (Hemiptera). The new beetle records included one specimen sampled of *Lagriahirta* (Linnaeus, 1758), eight of *Ischnopterapionvirens* (Herbst, 1797) and six of *Microlestesnegritanegrita* (Wollaston, 1854). All these individuals were collected in maize fields. The new millipede record included three specimens of *Nopoiuluskochii* (Gervais, 1847), also collected in maize fields, but at low elevation. Finally, the new hemipteran record included three specimens of *Cicadellaviridis* (Linnaeus, 1758) from a citrus orchard. All new records belong to introduced species, with the exception of *M.negritanegrita*, which is native to the Azores.

*Lagriahirta* (Coleoptera, Tenebrionidae) is a new record for Azores. We have also recently sampled this species in the Island of Santa Maria. This seems to be a recent introduction in Azores, being still rare in Terceira, but already widespread in Santa Maria.

### Future perspectives

Importantly, the EU Biodiversity Strategy 2020 lists, as a priority, the mapping and assessment of the state of biodiversity, ecosystems and their services in all EU member states (Maes et al. 2016). Azores are part of Europe’s nine Outermost Regions (ORs) for which there is a general lack of ES mapping and assessment as compared with mainland Europe (Sieber et al. 2018).

By focusing on Azorean Island agroecosystems (e.g. maize fields, vineyards, citrus orchards) and having the current baseline monitoring data, we aim to develop in the near future a multifaceted approach to gain more insight to evaluate the relative importance of native and exotic arthropod organisms as ecosystem services (ES)/ ecosystem disservices (ED) providers. In this way, it will be possible to understand the ecosystem processes and functions and the goods and services arthropods provide for improving the resilience of Azorean agro-ecosystems, as well as human well-being.

## Supplementary Material

811A3607-237B-58D4-9713-A945B378D866 10.3897/BDJ.9.e77548.suppl1 Supplementary material 1 Complete list of sampled species and mophospecies Data type Occurrences Brief description Detailed complete list of sampled species and mophospecies with indication of the morphospecies codes in the column (Identification Remarks) File: oo_611247.xlsx https://binary.pensoft.net/file/611247 Paulo A. V. Borges

## Figures and Tables

**Table 1.** Description of the habitat, locality, elevation and coordinates of the 18 sampled sites on Terceira Island, Azores.

Code Site	Habitat	Location ID	Locality	Elevation (m a.s.l.)	Latitude	Longitude
C1	Citrus	TER_CITRUS_T1_T206	Pico da Urze	117	38.66989	-27.24047
C2	Citrus	TER_CITRUS_T2_T207	Qt. Rosário	158	38.68111	-27.26206
C3	Citrus	TER_CITRUS_T3_T208	S. Bartolomeu	189	38.6827	-27.27555
C4	Citrus	TER_CITRUS_T4_T209	S. Bento	66	38.66287	-27.21019
C5	Citrus	TER_CITRUS_T5_T210	S. Carlos	69	38.6625	-27.24961
ML1	Maize Low	TER_MAIZE_LOW_T2_T221	Atalaia	111	38.65631	-27.18368
ML2	Maize Low	TER_MAIZE_LOW_T1_T220	Cinco Ribeiras	90	38.6758	-27.30998
ML3	Maize Low	TER_MAIZE_LOW_T3_T222	S. Mateus	42	38.66304	-27.28962
ML4	Maize Low	TER_MAIZE_LOW_T4_T223	Universidade dos Açores - Campus do Pico da Urze	36	38.659	-27.23555
ML5	Maize Low	TER_MAIZE_LOW_T5_T224	Vinha Brava	167	38.67593	-27.21684
MH1	Maize High	TER_MAIZE_HIGH_T1_T215	Casa da Mina	314	38.68602	-27.1974
MH2	Maize High	TER_MAIZE_HIGH_T2_T216	Escampadouro	309	38.70159	-27.2852
MH3	Maize High	TER_MAIZE_HIGH_T3_T217	Granja	385	38.70083	-27.17019
MH4	Maize High	TER_MAIZE_HIGH_T4_T218	Juncal	321	38.69996	-27.12048
MH5	Maize High	TER_MAIZE_HIGH_T5_T219	Poejo	275	38.6768	-27.14616
V1	Vineyards	TER_VINE_F1_T211	Biscoitos Vinha_F1	23	38.79793	-27.25567
V2	Vineyards	TER_VINE_F2_T212	Biscoitos Vinha_F2	52	38.79664	-27.26302
V3	Vineyards	TER_VINE_F3_T213	Biscoitos Vinha_F3	28	38.80066	-27.26842

**Table 2.** Inventory of arthropods collected in four agroecosystems in Terceira Island (Azores, Portugal) following an elevation gradient: vineyards (Vine), citrus orchards (Citrus), maize fields at low elevation (Maize L) and at high elevation (Maize H). The list includes only the specimens identified at species-level. Class, order, family, scientific name follow alphabetical sequence. Colonisation status based on Borges et al. 2010 (Origin: END - Endemic; NAT - native non-endemic; INTR - introduced) and abundance per habitat type are provided. Bold scientific names constitute new records for Terceira Island. * - New record for Azores.

class	order	family	scientificName	Origin	VINE	CITRUS	MAIZE L	MAIZE H	Total
Arachnida	Araneae	Agelenidae	*Tegenariadomestica* (Clerck, 1757)	INTR				1	1
Arachnida	Araneae	Agelenidae	*Tegenariapagana* C.L. Koch, 1840	INTR		3			3
Arachnida	Araneae	Araneidae	*Agalenatearedii* (Scopoli, 1763)	INTR			7	2	9
Arachnida	Araneae	Araneidae	*Araneusangulatus* Clerck, 1757	INTR			30		30
Arachnida	Araneae	Araneidae	*Argiopebruennichi* (Scopoli, 1772)	NAT			37	50	87
Arachnida	Araneae	Araneidae	*Gibbaraneaoccidentalis* Wunderlich, 1989	END				1	1
Arachnida	Araneae	Araneidae	*Mangoraacalypha* (Walckenaer, 1802)	INTR				1	1
Arachnida	Araneae	Araneidae	*Neosconacrucifera* (Lucas, 1838)	INTR			2	2	4
Arachnida	Araneae	Araneidae	*Zygiellax-notata* (Clerck, 1757)	INTR			6	12	18
Arachnida	Araneae	Clubionidae	*Clubionaterrestris* Westring, 1851	INTR		2			2
Arachnida	Araneae	Clubionidae	*Porrhoclubionadecora* (Blackwall, 1859)	NAT			25	4	29
Arachnida	Araneae	Clubionidae	*Porrhoclubionagenevensis* (L. Koch, 1866)	INTR			1		1
Arachnida	Araneae	Dictynidae	*Lathysdentichelis* (Simon, 1883)	NAT		1			1
Arachnida	Araneae	Dictynidae	*Nigmapuella* (Simon, 1870)	INTR			3		3
Arachnida	Araneae	Dysderidae	*Dysderacrocata* C.L. Koch, 1838	INTR	4	70	20	15	109
Arachnida	Araneae	Gnaphosidae	*Marinarozeloteslyonneti* (Audouin, 1826)	INTR	15		15		30
Arachnida	Araneae	Linyphiidae	*Agynetadecora* (O. Pickard-Cambridge, 1871)	INTR			1		1
Arachnida	Araneae	Linyphiidae	*Agynetafuscipalpa* (C. L. Koch, 1836)	INTR	28	7	396	18	449
Arachnida	Araneae	Linyphiidae	*Erigoneatra* Blackwall, 1833	INTR	1	3	3	13	20
Arachnida	Araneae	Linyphiidae	*Erigoneautumnalis* Emerton, 1882	INTR	1	309	333	95	738
Arachnida	Araneae	Linyphiidae	*Erigonedentipalpis* (Wider, 1834)	INTR		2	176	484	662
Arachnida	Araneae	Linyphiidae	*Mermessusbryantae* (Ivie & Barrows, 1935)	INTR		2	3	2	7
Arachnida	Araneae	Linyphiidae	*Mermessusfradeorum* (Berland, 1932)	INTR		117	7	53	177
Arachnida	Araneae	Linyphiidae	*Nerieneclathrat*a (Sundevall, 1830)	INTR		3	2	2	7
Arachnida	Araneae	Linyphiidae	*Oedothoraxfuscus* (Blackwall, 1834)	INTR		4	80	577	661
Arachnida	Araneae	Linyphiidae	*Osteariusmelanopygius* (O. Pickard-Cambridge, 1880)	INTR		1	6	17	24
Arachnida	Araneae	Linyphiidae	*Palliduphantesschmitzi* (Kulczynski, 1899)	NAT	7	1	1	2	11
Arachnida	Araneae	Linyphiidae	*Pelecopsisparallela* (Wider, 1834)	INTR	32		1		33
Arachnida	Araneae	Linyphiidae	*Prinerigonevagans* (Audouin, 1826)	INTR			130	229	359
Arachnida	Araneae	Linyphiidae	*Tenuiphantestenui*s (Blackwall, 1852)	INTR		132	104	177	413
Arachnida	Araneae	Lycosidae	*Arctosaperita* (Latreille, 1799)	INTR			1		1
Arachnida	Araneae	Lycosidae	*Pardosaacorensis* Simon, 1883	END		6		3	9
Arachnida	Araneae	Oecobiidae	*Oecobiusnavus* Blackwall, 1859	INTR	5		5		10
Arachnida	Araneae	Salticidae	*Chalcoscirtusinfimus* (Simon, 1868)	INTR	14				14
Arachnida	Araneae	Salticidae	*Heliophanuskochii* Simon, 1868	INTR		1			1
Arachnida	Araneae	Salticidae	*Macaroerisdiligens* (Blackwall, 1867)	NAT			1	2	3
Arachnida	Araneae	Salticidae	*Pseudeuophrysvafra* (Blackwall, 1867)	INTR	3				3
Arachnida	Araneae	Salticidae	*Salticusmutabilis* Lucas, 1846	INTR			1		1
Arachnida	Araneae	Salticidae	*Synagelesvenator* (Lucas, 1836)	INTR		1			1
Arachnida	Araneae	Scytotidae	*Scytodesthoracica* (Latreille, 1802)	INTR			1		1
Arachnida	Araneae	Segestriidae	*Segestriaflorentina* (Rossi, 1790)	INTR				1	1
Arachnida	Araneae	Tetragnathidae	*Pachygnathadegeeri* Sundevall, 1830	INTR			1	55	56
Arachnida	Araneae	Theridiidae	*Cryptachaeablattea* (Urquhart, 1886)	INTR		5	2	11	18
Arachnida	Araneae	Theridiidae	*Neottiurabimaculata* (Linnaeus, 1767)	INTR	1				1
Arachnida	Araneae	Theridiidae	*Parasteatodatepidariorum* (C. L. Koch, 1841)	INTR			8	69	77
Arachnida	Araneae	Theridiidae	*Steatodagrossa* (C. L. Koch, 1838)	INTR			16	71	87
Arachnida	Araneae	Theridiidae	*Steatodanobilis* (Thorell, 1875)	INTR				2	2
Arachnida	Araneae	Theridiidae	*Theridionmelanostictum* O. Pickard-Cambridge, 1876	INTR		1	3		4
Arachnida	Araneae	Theridiidae	*Theridionmusivivum* Schmidt, 1956	NAT		1			1
Arachnida	Araneae	Thomisidae	*Xysticusnubilus* Simon, 1875	INTR			3		3
Arachnida	Araneae	Zodariidae	*Zodarionatlanticum* Pekár & Cardoso, 2005	INTR	934	7	14	1	956
Arachnida	Opiliones	Phalangiidae	*Homalenotuscoriaceus* (Simon, 1879)	NAT	1	156		20	177
Arachnida	Opiliones	Phalangiidae	*Leiobunumblackwalli* Meade, 1861	NAT		7		12	19
Arachnida	Pseudoscorpiones	Chthoniidae	*Chthoniusischnocheles* (Hermann, 1804)	INTR	8	10	4		22
Arachnida	Pseudoscorpiones	Chthoniidae	*Ephippiochthoniustetrachelatus* (Preyssler, 1790)	INTR	18	9			27
Arachnida	Pseudoscorpiones	Neobisiidae	*Neobisiummaroccanum* Beier, 1930	INTR	1	2			3
Chilopoda	Geophilomorpha	Linotaeniidae	*Strigamiacrassipes* (C.L. Koch, 1835)	NAT		2			2
Chilopoda	Lithobiomorpha	Lithobiidae	*Lithobiuspilicornispilicornis* Newport, 1844	NAT	15	4	1	1	21
Chilopoda	Scolopendromorpha	Cryptopidae	*Cryptopshortensis* (Donovan, 1810)	NAT	6	1	2		9
Chilopoda	Scutigeromorpha	Scutigeridae	*Scutigeracoleoptrata* (Linnaeus, 1758)	INTR	34	205	171	27	437
Diplopoda	Chordeumatida	Haplobainosomatidae	*Haplobainosomalusitanum* Verhoeff, 1900	INTR		6			6
Diplopoda	Julida	Blaniulidae	*Blaniulusguttulatus* (Fabricius, 1798)	INTR		1			1
Diplopoda	Julida	Blaniulidae	***Nopoiuluskochii* (Gervais, 1847)**	INTR			3		3
Diplopoda	Julida	Blaniulidae	*Proteroiulusfuscus* (Am Stein, 1857)	INTR		3			3
Diplopoda	Julida	Julidae	*Brachyiuluspusillus* (Leach, 1814)	INTR		138			138
Diplopoda	Julida	Julidae	*Cylindroiuluslatestriatus* (Curtis, 1845)	INTR		1			1
Diplopoda	Julida	Julidae	*Cylindroiuluspropinquus* (Porat, 1870)	INTR	4	14			18
Diplopoda	Julida	Julidae	*Ommatoiulusmoreleti* (Lucas, 1860)	INTR	221	1740	35	217	2213
Diplopoda	Polydesmida	Polydesmidae	*Brachydesmussuperus* Latzel, 1884	INTR		1			1
Diplopoda	Polydesmida	Polydesmidae	*Polydesmuscoriaceus* Porat, 1870	INTR	8	470	12	53	543
Insecta	Archaeognatha	Machilidae	*Diltasaxicola* (Womersley, 1930)	NAT			3	4	7
Insecta	Coleoptera	Anthicidae	*Hirticollisquadriguttatus* (Rossi, 1792)	NAT	1		166	176	343
Insecta	Coleoptera	Apionidae	*Aspidapionradiolus* (Marsham, 1802)	NAT	1		1		2
Insecta	Coleoptera	Apionidae	***Ischnopterapionvirens* (Herbst, 1797)**	INTR			6	2	8
Insecta	Coleoptera	Carabidae	*Acupalpusdubius* Schilsky, 1888	NAT			37	8	45
Insecta	Coleoptera	Carabidae	*Acupalpusflavicollis* (Sturm, 1825)	NAT			47	1	48
Insecta	Coleoptera	Carabidae	*Agonummuellerimuelleri* (Herbst, 1784)	INTR				38	38
Insecta	Coleoptera	Carabidae	*Amaraaenea* (De Geer, 1774)	INTR		1	6	15	22
Insecta	Coleoptera	Carabidae	*Anisodactylusbinotatus* (Fabricius, 1787)	INTR		1	3	65	69
Insecta	Coleoptera	Carabidae	*Calosomaolivieri* Dejean, 1831	NAT			14	41	55
Insecta	Coleoptera	Carabidae	*Harpalusdistinguendusdistinguendus* (Duftschmid, 1812)	INTR		1	3	40	44
Insecta	Coleoptera	Carabidae	*Laemostenuscomplanatus* (Dejean, 1828)	INTR	5	41		1	47
Insecta	Coleoptera	Carabidae	***Microlestesnegritanegrita* (Wollaston, 1854)**	NAT			6		6
Insecta	Coleoptera	Carabidae	*Notiophilusquadripunctatus* Dejean, 1826	NAT				1	1
Insecta	Coleoptera	Carabidae	*Ocysharpaloides* (Audinet-Serville, 1821)	NAT		5			5
Insecta	Coleoptera	Carabidae	*Paranchusalbipes* (Fabricius, 1796)	INTR		1		16	17
Insecta	Coleoptera	Carabidae	*Pseudoophonusrufipes* (De Geer, 1774)	INTR	7	74	55	6995	7131
Insecta	Coleoptera	Carabidae	*Pterostichusvernalis* (Panzer, 1796)	INTR				25	25
Insecta	Coleoptera	Chrysomelidae	*Chaetocnemahortensis* (Fourcroy, 1785)	INTR		1	2		3
Insecta	Coleoptera	Chrysomelidae	*Chrysolinabankii* (Fabricius, 1775)	NAT		10			10
Insecta	Coleoptera	Chrysomelidae	*Epitrixcucumeris* (Harris, 1851)	INTR	53	4			57
Insecta	Coleoptera	Chrysomelidae	*Longitarsuskutscherai* (Rye, 1872)	INTR			1		1
Insecta	Coleoptera	Coccinellidae	*Scymniscushelgae* (Fürsch, 1965)	INTR		1			1
Insecta	Coleoptera	Corylophidae	*Sericoderuslateralis* (Gyllenhal, 1827)	INTR	15	61	268	96	440
Insecta	Coleoptera	Curculionidae	*Calacallessubcarinatus* (Israelson, 1984)	END		1			1
Insecta	Coleoptera	Curculionidae	*Cathormioceruscurvipes* (Wollaston, 1854)	NAT		18			18
Insecta	Coleoptera	Curculionidae	*Coccotrypescarpophagus* (Hornung, 1842)	INTR		71	3	2	76
Insecta	Coleoptera	Curculionidae	*Naupactuscervinus* (Boheman, 1840)	INTR		4			4
Insecta	Coleoptera	Curculionidae	*Orthochaetesinsignis* (Aubé, 1863)	NAT	1	21			22
Insecta	Coleoptera	Curculionidae	*Otiorhynchuscribricollis* Gyllenhal, 1834	INTR		5			5
Insecta	Coleoptera	Curculionidae	*Otiorhynchusrugosostriatus* (Goeze, 1777)	INTR	4	1			5
Insecta	Coleoptera	Curculionidae	*Pseudophloeophagustenax* Wollaston, 1854	NAT		2			2
Insecta	Coleoptera	Curculionidae	*Xyleborinusalni* Nijima, 1909	INTR				1	1
Insecta	Coleoptera	Dryophthoridae	*Cosmopolitessordidu*s (Germar, 1824)	INTR		1			1
Insecta	Coleoptera	Dryophthoridae	*Sphenophorusabbreviatus* (Fabricius, 1787)	INTR		4	2	51	57
Insecta	Coleoptera	Elateridae	*Aeolusmelliculusmoreleti* Tarnier, 1860	INTR			8		8
Insecta	Coleoptera	Elateridae	*Heteroderesazoricus* (Tarnier, 1860)	END			2	1	3
Insecta	Coleoptera	Elateridae	*Heteroderesvagus* Candèze, 1893	INTR			3	13	16
Insecta	Coleoptera	Elateridae	*Melanotusdichrous* (Erichson, 1841)	INTR			14		14
Insecta	Coleoptera	Histeridae	*Carcinopspumilio* (Erichson, 1834)	INTR	1				1
Insecta	Coleoptera	Hydrophilidae	*Sphaeridiumbipustulatum* Fabricius, 1781	INTR			1	1	2
Insecta	Coleoptera	Latridiidae	*Cartoderenodifer* (Westwood, 1839)	INTR		2	1		3
Insecta	Coleoptera	Leiodidae	*Catopscoracinus* Kellner, 1846	NAT			1		1
Insecta	Coleoptera	Malachiidae	*Attaluslusitanicuslusitanicus* Erichson, 1840	NAT			2		2
Insecta	Coleoptera	Mycetophagidae	*Litargusbalteatus* Le Conte, 1856	INTR		1		1	2
Insecta	Coleoptera	Mycetophagidae	*Typhaeastercorea* (Linnaeus, 1758)	INTR	1		642	5	648
Insecta	Coleoptera	Nitidulidae	*Carpophilusfumatus* Boheman, 1851	INTR		1			1
Insecta	Coleoptera	Nitidulidae	*Epuraeabiguttata* (Thunberg, 1784)	INTR	49	22		1	72
Insecta	Coleoptera	Nitidulidae	*Phenolialimbatatibialis* (Boheman, 1851)	INTR	15	6	1	1	23
Insecta	Coleoptera	Nitidulidae	*Stelidotageminata* (Say, 1825)	INTR			128	18	146
Insecta	Coleoptera	Phalacridae	*Stilbustestaceus* (Panzer, 1797)	NAT		1	24	1	26
Insecta	Coleoptera	Ptiliidae	*Ptenidiumpusillum* (Gyllenhal, 1808)	INTR	4	6	2		12
Insecta	Coleoptera	Scarabaeidae	*Calamosternusgranarius* (Linnaeus, 1767)	INTR			7		7
Insecta	Coleoptera	Scarabaeidae	*Onthophagusvacca* (Linnaeus, 1767)	INTR				6	6
Insecta	Coleoptera	Scarabaeidae	*Popilliajaponica* Newman, 1838	INTR				4	4
Insecta	Coleoptera	Silvanidae	*Cryptamorphadesjardinsii* (Guérin-Méneville, 1844)	INTR		3			3
Insecta	Coleoptera	Staphylinidae	*Aleocharabipustulata* (Linnaeus, 1760)	INTR	1		1	4	6
Insecta	Coleoptera	Staphylinidae	*Aloconotasulcifrons* (Stephens, 1832)	NAT			11		11
Insecta	Coleoptera	Staphylinidae	*Amischaanalis* (Gravenhorst, 1802)	INTR	1	8	48	1321	1378
Insecta	Coleoptera	Staphylinidae	*Anotylusnitidifrons* (Wollaston, 1871)	INTR	10	377	4	8	399
Insecta	Coleoptera	Staphylinidae	*Anotylusnitidulus* (Gravenhorst, 1802)	INTR		2			2
Insecta	Coleoptera	Staphylinidae	*Astenuslyonessius* (Joy, 1908)	NAT			10		10
Insecta	Coleoptera	Staphylinidae	*Athetaaeneicollis* (Sharp, 1869)	INTR	1	2			3
Insecta	Coleoptera	Staphylinidae	*Athetafungi* (Gravenhorst, 1806)	INTR	1	76	66	49	192
Insecta	Coleoptera	Staphylinidae	*Carpelimuscorticinus* (Gravenhorst, 1806)	NAT		1			1
Insecta	Coleoptera	Staphylinidae	*Coproporuspulchellu*s (Erichson, 1839)	INTR		6			6
Insecta	Coleoptera	Staphylinidae	*Cordaliaobscura* (Gravenhorst, 1802)	INTR	20	17	256	316	609
Insecta	Coleoptera	Staphylinidae	*Euplectusinfirmus* Raffray, 1910	INTR	1	2			3
Insecta	Coleoptera	Staphylinidae	*Gabriusnigritulus* (Gravenhorst, 1802)	INTR			2	3	5
Insecta	Coleoptera	Staphylinidae	*Medonapicalis* (Kraatz, 1857)	NAT		1			1
Insecta	Coleoptera	Staphylinidae	*Ocypusaethiops* (Waltl, 1835)	NAT		308		1	309
Insecta	Coleoptera	Staphylinidae	*Ocypusolens* (Müller, 1764)	NAT		59		45	104
Insecta	Coleoptera	Staphylinidae	*Oligotapumilio* Kiesenwetter, 1858	NAT	7	70	178	12	267
Insecta	Coleoptera	Staphylinidae	*Phloeonomuspunctipennis* Thomson, 1867	NAT		1			1
Insecta	Coleoptera	Staphylinidae	*Proteinusatomarius* Erichson, 1840	NAT		10			10
Insecta	Coleoptera	Staphylinidae	*Pseudoplectusperplexus* (Jacquelin du Val, 1854)	NAT	22	4		41	67
Insecta	Coleoptera	Staphylinidae	*Quediuscurtipennis* Bernhauer, 1908	NAT				1	1
Insecta	Coleoptera	Staphylinidae	*Rugilusorbiculatus* (Paykull, 1789)	NAT		2	365	757	1124
Insecta	Coleoptera	Staphylinidae	*Sepedophiluslusitanicus* Hammond, 1973	NAT		4			4
Insecta	Coleoptera	Staphylinidae	*Stenomastaxmaderae* Assing, 2003	NAT		127			127
Insecta	Coleoptera	Staphylinidae	*Tachyporuschrysomelinus* (Linnaeus, 1758)	INTR	1				1
Insecta	Coleoptera	Staphylinidae	*Tachyporusnitidulus* (Fabricius, 1781)	INTR	1	2	5	3	11
Insecta	Coleoptera	Staphylinidae	*Trichiusaimmigrata* Lohse, 1984	INTR	3				3
Insecta	Coleoptera	Staphylinidae	*Xantholinuslongiventris* Heer, 1839	INTR			3	1	4
Insecta	Coleoptera	Tenebrionidae	*Blaps lethifera* Marsham, 1802	INTR			1		1
Insecta	Coleoptera	Tenebrionidae	*Lagriahirta* (Linnaeus, 1758)*	INTR			1		1
Insecta	Dermaptera	Anisolabididae	*Euborelliaannulipes* (Lucas, 1847)	INTR	2	116		26	144
Insecta	Dermaptera	Forficulidae	*Forficulaauricularia* Linnaeus, 1758	INTR		2	155	232	389
Insecta	Hemiptera	Anthocoridae	*Anthocorisnemoralis* (Fabricius, 1794)	NAT			1		1
Insecta	Hemiptera	Anthocoridae	*Oriuslaevigatuslaevigatus* (Fieber, 1860)	NAT			1		1
Insecta	Hemiptera	Aphididae	*Rhopalosiphoninuslatysiphon* (Davidson, 1912)	INTR	6	43			49
Insecta	Hemiptera	Cicadellidae	*Anoscopusalbifrons* (Linnaeus, 1758)	NAT	1	3	6		10
Insecta	Hemiptera	Cicadellidae	***Cicadellaviridis* (Linnaeus, 1758)**	INTR		3			3
Insecta	Hemiptera	Cicadellidae	*Euscelidiusvariegatus* (Kirschbaum, 1858)	NAT			72	10	82
Insecta	Hemiptera	Cicadellidae	*Sophoniaorientalis* (Matsumura, 1912)	INTR		1			1
Insecta	Hemiptera	Cydnidae	*Geotomuspunctulatus* (A. Costa, 1847)	NAT	33	3	3	1	40
Insecta	Hemiptera	Delphacidae	*Kelisiaribauti* Wagner, 1938	NAT		8	41	116	165
Insecta	Hemiptera	Delphacidae	*Megamelodesquadrimaculatus* (Signoret, 1865)	NAT		1			1
Insecta	Hemiptera	Lygaeidae	*Aphanusrolandri* (Linnaeus, 1758)	NAT	7		3		10
Insecta	Hemiptera	Lygaeidae	*Heterogasterurticae* (Fabricius, 1775)	NAT			1		1
Insecta	Hemiptera	Lygaeidae	*Kleidoceryserica*e (Horváth, 1909)	NAT	1				1
Insecta	Hemiptera	Lygaeidae	*Oxycarenuslavaterae* (Fabricius, 1787)	INTR			1		1
Insecta	Hemiptera	Lygaeidae	*Scolopostethusdecoratu*s (Hahn, 1833)	NAT	6	33	1	1	41
Insecta	Hemiptera	Microphysidae	*Loriculaelegantula* (Bärensprung, 1858)	NAT		1			1
Insecta	Hemiptera	Miridae	*Campyloneuravirgula* (Herrich-Schaeffer, 1835)	NAT				1	1
Insecta	Hemiptera	Miridae	*Heterotomaplanicornis* (Pallas, 1772)	NAT			4		4
Insecta	Hemiptera	Miridae	*Pilophorusconfusus* (Kirschbaum, 1856)	NAT			1		1
Insecta	Hemiptera	Miridae	*Trigonotyluscaelestialium* (Kirkaldy, 1902)	NAT			493	231	724
Insecta	Hemiptera	Nabidae	*Nabispseudoferusibericus* Remane, 1962	NAT			7	46	53
Insecta	Hemiptera	Pentatomidae	*Nezaraviridula* (Linnaeus, 1758)	INTR			5	6	11
Insecta	Hemiptera	Reduviidae	*Empicorisrubromaculatus* (Blackburn, 1889)	INTR		10	1		11
Insecta	Hemiptera	Reduviidae	*Ploiariadomestica* Scopoli, 1786	INTR			1		1
Insecta	Hemiptera	Saldidae	*Saldulapalustris* (Douglas, 1874)	NAT				1	1
Insecta	Hemiptera	Tingidae	*Acalyptaparvula* (Fallén, 1807)	NAT	5	4			9
Insecta	Hymenoptera	Apidae	*Bombusterrestris* (Linnaeus, 1758)	INTR			1	1	2
Insecta	Hymenoptera	Formicidae	*Hypoponeraeduardi* (Forel, 1894)	NAT	12	32	37	99	180
Insecta	Hymenoptera	Formicidae	*Lasiusgrandis* Forel, 1909	NAT	10283	3058	1444	1091	15876
Insecta	Hymenoptera	Formicidae	*Linepithemahumile* (Mayr, 1868)	INTR			2		2
Insecta	Hymenoptera	Formicidae	*Monomoriumcarbonarium* (Smith, 1858)	NAT	272	367		1	640
Insecta	Hymenoptera	Formicidae	*Tetramoriumcaespitum* (Linnaeus, 1758)	NAT	327	1329	1202	451	3309
Insecta	Hymenoptera	Formicidae	*Tetramoriumcaldarium* (Roger, 1857)	INTR	215	135	1		351
Insecta	Lepidoptera	Noctuidae	*Mythimnaunipuncta* (Haworth, 1809)	NAT				1	1
Insecta	Orthoptera	Gryllidae	*Eumodicogryllusbordigalensis* (Latreille, 1804)	INTR	1	1		1559	1561
Insecta	Orthoptera	Gryllidae	*Gryllusbimaculatus* De Geer, 1773	INTR		10			10
Insecta	Orthoptera	Phaneropteridae	*Phaneropteranana* Fieber, 1853	NAT				2	2
Insecta	Psocoptera	Caeciliusidae	*Valenzuelaflavidus* (Stephens, 1836)	NAT	1		27	1	29
Insecta	Psocoptera	Ectopsocidae	*Ectopsocusbriggsi* McLachlan, 1899	INTR		1	28	18	47
Insecta	Psocoptera	Ectopsocidae	*Ectopsocusstrauchi* Enderlein, 1906	NAT	1				1
Insecta	Psocoptera	Trichopsocidae	*Trichopsocusclarus* (Banks, 1908)	NAT		2			2
Insecta	Thysanoptera	Thripidae	*Hercinothripsbicinctus* (Bagnall, 1919)	INTR	3			1	4
			**Grand Total**		**12763**	**10062**	**7622**	**16390**	**46837**
